# The Feasibility of Embedding Data Collection into the Routine Service Delivery of a Multi-Component Program for High-Risk Young People

**DOI:** 10.3390/ijerph14020208

**Published:** 2017-02-20

**Authors:** Alice Knight, Alys Havard, Anthony Shakeshaft, Myfanwy Maple, Mieke Snijder, Bernie Shakeshaft

**Affiliations:** 1 National Drug and Alcohol Research Centre (NDARC), University of New South Wales, Randwick, NSW 2052, Australia; alys.havard@unsw.edu.au (A.H.); a.shakeshaft@unsw.edu.au (A.S.); m.snijder@unsw.edu.au (M.S.); 2 Centre for Big Data Research in Health, University of New South Wales, Randwick, NSW 2052, Australia; 3 School of Health, University of New England, Armidale, NSW 2351, Australia; mmaple2@une.edu.au; 4 BackTrack, Armidale, NSW 2350, Australia; bernie@backtrack.org.au

**Keywords:** high-risk young people, Indigenous, male, community-based program, embedded research, co-created research

## Abstract

Background: There is little evidence about how to improve outcomes for high-risk young people, of whom Indigenous young people are disproportionately represented, due to few evaluation studies of interventions. One way to increase the evidence is to have researchers and service providers collaborate to embed evaluation into the routine delivery of services, so program delivery and evaluation occur simultaneously. This study aims to demonstrate the feasibility of integrating best-evidence measures into the routine data collection processes of a service for high-risk young people, and identify the number and nature of risk factors experienced by participants. Methods: The youth service is a rural based NGO comprised of multiple program components: (i) engagement activities; (ii) case management; (iii) diversionary activities; (iv) personal development; and (v) learning and skills. A best-evidence assessment tool was developed by staff and researchers and embedded into the service’s existing intake procedure. Assessment items were organised into demographic characteristics and four domains of risk: education and employment; health and wellbeing; substance use; and crime. Descriptive data are presented and summary risk variables were created for each domain of risk. A count of these summary variables represented the number of co-occurring risks experienced by each participant. The feasibility of this process was determined by the proportion of participants who completed the intake assessment and provided research consent. Results: This study shows 85% of participants completed the assessment tool demonstrating that data on participant risk factors can feasibly be collected by embedding a best-evidence assessment tool into the routine data collection processes of a service. The most prevalent risk factors were school absence, unemployment, suicide ideation, mental distress, substance use, low levels of physical activity, low health service utilisation, and involvement in crime or with the juvenile justice system. All but one participant experienced at least two co-occurring domains of risk, and the majority of participants (58%) experienced co-occurring risk across four domains. Conclusions: This is the first study to demonstrate that best-evidence measures can feasibly be embedded into the routine data collection processes of a service for high-risk young people. This process allows services to tailor their activities to the most prevalent risks experienced by participants, and monitor these risks over time. Replication of this process in other services would improve the quality of services, facilitate more high quality evaluations of services, and contribute evidence on how to improve outcomes for high-risk young people.

## 1. Introduction

Despite being experienced as a turbulent time most young people successfully navigate adolescence and avoid any serious, long-term harms. A minority of young people, however, will experience more substantial harms associated with co-occurring risk-factors (high-risk young people), including substance abuse, low engagement in education and employment, exposure to violence and/or suicide, homelessness, participation in criminal activity, and/or incarceration [1,2,3]. Moreover, the presence of co-occurring risk factors has a multiplier effect, even if the causal relationship between these factors is unclear: increased frequency of substance abuse and involvement in crime, for example, is associated with disengagement from school and declining academic performance, which in turn is associated with reduced employment opportunities (as a consequence of criminal convictions and poor literacy) and increased risk of self-harm, suicide and recidivism into both juvenile and adult prisons [4,5,6,7,8].

Complicating the presence of co-occurring risk factors is that their aetiology is typically complex, being associated with a range of social determinants of poor health, including childhood abuse, low socio-economic status (SES) and minority cultural identity [9,10,11]. This complex aetiology implies that the occurrence of significant harm among young people will not be spread randomly across individuals in a population, but will cluster within defined sub-populations. Indigenous people in Australia, for example, have had a recent history of dispossession, racism, oppression and low socio-economic status which, in turn, has increased the rate with which they experience mental health and physical harms: alcohol-related suicide rates among 15–29 year old Indigenous Australians are four (males) and five (females) times higher than for non-Indigenous young people [12]; rates of all-cause alcohol-related disease and injury are more than double for Indigenous males and seven times greater for Indigenous females [12]; and more than 50% of 10–17 year old juvenile detainees are Indigenous, despite Indigenous Australians comprising an estimated 2% of the population [13,14].

Despite this harm, very little is known about ways of effectively intervening with high-risk young people: a current systematic review found only 10% (n = 13) of 129 evaluation studies of interventions for high-risk young people were for interventions that addressed co-occurring risk factors and more than half of the studies (n = 7) were rated as methodologically weak [15]. This finding mirrors those from other fields engaged in complex social or health interventions, and is likely a direct result of the complexity, cost and time involved in evaluating these types of programs, and that the focus of most service staff is on the day-to-day delivery of their program, not conducting scientifically rigorous evaluation [16,17,18].

One way to increase the number and quality of evaluations of services for high-risk young people is to have researchers and service providers collaborate on developing evaluations that can be embedded into the routine delivery of services so that program delivery and evaluation occur simultaneously, a process described as co-creation [19] or co-production [20]. Embedded evaluations would ideally integrate best-evidence measures into the routine data collection processes of services which, in addition to facilitating evaluation of effectiveness, would improve the accuracy with which risk factors experienced by high-risk young people accessing services are identified, allowing organisations to improve their efforts to tailor services to the specific needs of participants. Repeated application of best-evidence measures would have the added benefit of providing services with the ability to monitor the changing needs of their participants over time so they can modify their program components accordingly.

Despite the potential benefits of co-creation, services for high-risk young people do not appear to be embedding data collection into routine service delivery: outcome data for all 13 evaluations identified in a recent systematic review were collected by members of a research team, as opposed to being embedded into the routine data collection processes of the service [15]. In order to encourage greater embedded data collection in this field, this study aims to demonstrate the feasibility of integrating best-evidence measures into the routine data collection processes of a service for high-risk young people, and identify the number and nature of risk factors experienced by the service participants.

## 2. Methods

### 2.1. Service and Setting

The service is a Non-Government Organisation (NGO), established in 2006 in a rural community in the New England region of New South Wales (NSW), Australia. Its broad objective is to provide alternative and positive pathways into adulthood for high-risk young people by providing a multi-component service that can target co-occurring risk factors. This objective is operationalised through a number of flexible activities, organised into five standardised core program components: (i) *effective engagement*, to optimise participation in the program; (ii) *case management*, to address participants’ immediate and practical needs, such as attending court or homelessness; (iii) *diversionary activities*, to reduce participants’ exposure to high-risk situations, such as night-time encounters with police in public places or volatile situations at home; (iv) *personal development, identity and team identity*, to improve participants’ personal coping strategies when they are in high-risk situations and their sense of connection to their peers and community; and (v) *learning and skills*, to increase their opportunities for active participation in education or training likely to lead to employment.

The model of standardisation (the five core program components) with built-in flexibility (the specific activities that operationalise each component which are selected and designed by staff) provides a mechanism to both standardise the intervention across multiple communities and tailor it to the resources available in different communities. This service has been implemented in different formats for high-risk young people in five different communities. For the first community, the service was delivered in a previously disused shed donated by the local council. For the second and third communities, services were provided as an outreach model through a combination of young people attending the shed in the first community, and staff from the first community providing activities in the second and third communities. For the fourth and fifth communities, high-risk young people accessed the service on a working, but largely disused, farm.

For the purpose of this paper, the five communities were clustered into three groups based on the different service delivery models: on-site based in a shed (community one); outreach (communities two and three); and on-site based on a farm (communities four and five).

### 2.2. Participants of the Service

Young people are eligible to participate in the service if they: (i) reside in a community where the service is available; (ii) are aged 14–21 years; and (iii) are currently experiencing more than one of the following behavioural risk factors: involvement in criminal activity; substance use; violent behaviour; homelessness; poor mental health and wellbeing; poor engagement with school (including suspensions and unexplained absences); and un- or under-employment.

Potential participants are referred from: individuals (self-referral, family members/primary caregivers, or a community member); local schools (because they are at risk of becoming completely and permanently disconnected from mainstream education); or another government or non-government agency (e.g., police, magistrate, NSW Department of Family and Community Services). Each referral is made using a standard expression of interest (EOI) form, comprising questions about the young person’s status in relation to the eligibility criteria. The EOI is reviewed by the program manager and at least two senior staff, each of whom provide a recommendation. The manager makes the final decision on placement. Young people who meet the inclusion criteria and are recommended for placement are then interviewed by senior staff. Those who demonstrate a commitment to personal growth and appear genuinely self-motivated to participate are invited to attend the program for one week on a trial basis. If the number of suitable referrals is greater than the places currently available in the program, they are placed on a waiting list. Those who do not meet the inclusion criteria, or are not invited for a trial placement, have this decision explained to them and their referring agent by a senior staff member in a face-to-face meeting, and they are given the option of being referred to a more appropriate agency.

Trial participants become program participants if they successfully complete their trial week and attend at least four days in the first month. Participants who leave the program are welcome to recommence when it suits them, and the program manager ensures there are vacancies in the program for this eventuality. This flexibility is designed to foster ownership of decisions and personal responsibility.

### 2.3. Measures

Prior to establishing the NGO and researcher partnership, the service’s intake procedure comprised the completion of a basic administrative form (e.g., emergency contact details), and the setting of priorities and goals for participants. Collaboration between service staff and the researchers resulted in the development of a new, practically relevant, and scientifically rigorous routine assessment tool that was embedded into the existing intake procedure to measure participants’ risk factors. Acknowledging the dearth of appropriate measures of risk factors with published evidence for their reliability and validity amongst young people, let alone high-risk young people [21], this collaboration achieved a compromise between pragmatism and scientific rigor by using “best-evidence” (BE) measures of risk factors. These are psychometrically tested measures with published evidence for their reliability or validity amongst a similar youth population, or where this is not possible, a normative adult population. Where these were unavailable, but staff required the information nonetheless for program design or monitoring of participant risk factors, non-psychometrically tested assessment items were sourced from surveys that targeted a similar population (SP) group (e.g., the NSW Schools Students Health Behaviours Survey [22]). Where BE or SP assessment items could not be identified, new (N) items were developed by the researchers in partnership with staff. Assessment items were organised into demographic characteristics and four domains of risk sourced from a classification developed by the authors in a previous systematic review of services for high-risk young people [15]:

*Demographic characteristics (SP):* Gender, date of birth, Aboriginal or Torres Strait Islander status, and community of residence.

*Education and employment (SP):* Number of school suspensions in the past six months, frequency of school attendance, employment status, and receipt of a government financial benefit [22].

*Mental health and wellbeing (BE, SP, and N):* In line with previous research [23], a summary measure of recent suicide ideation was based on positive endorsement of one or more of five yes/no items from the Psychiatric Symptom Frequency Scale [24]: “In the past four weeks have you ever felt that life is hardly worth living?”; “In the past four weeks have you ever thought that you would be better off dead?”; “In the past four weeks have you ever thought about taking your own life?”; “In the past four weeks have you ever made plans to take your own life?”; and “In the past four weeks have you ever attempted to take your own life?”. Given staff highlighted the importance of this measure as a screening tool to identify participants experiencing current suicide ideation, the time period from the original scale, which assesses suicide ideation “in the past year”, was modified to assess suicide ideation “in the past four weeks”. Resilience was measured using a brief 10-item version (CD-RISC-10) of the original 25-item Connor-Davidson resilience scale [25,26]. This reliable and valid scale assesses respondents’ perceptions of their ability to adapt to change, to deal with unexpected events, to cope with illness, injury, unpleasant feelings or obstacles, and to remain positive in stressful situations. The CD-RISC-10 was favoured by staff over the original version because it has been administered to youth samples [27,28] and its brevity reduces its response burden. Items are scored on a scale from 0 (not true at all) to 4 (true nearly all the time) and summed to a total score ranging from 0 to 40. Higher scores reflect greater resilience. Mental distress was measured using the six-item Kessler Psychological Distress Scale (K6), where each item was again rated on a five-point scale from 0 (none of the time) to 4 (all of the time) and summed to a total score from 0 to 24 [29,30]. A score of ≥5 indicates moderate mental distress and a score of ≥19 indicates serious mental distress.

General health and wellbeing items relating to frequency of fast food consumption in the past week and frequency of physical activity in the past week were sourced from the NSW Schools Students Health Behaviours Survey [22], and a new item was developed to measure frequency of health service utilisation.

*Substance use (BE and SP):* Risky drinking was measured using the AUDIT-C (comprising the first three items of the Alcohol Use Disorders Identification Test (AUDIT) [31]) which has demonstrable evidence for its reliability and validity, and performs well as an abbreviated alcohol screening measure in integrated health-risk surveys delivered in non-medical, community settings [32]. Given staff indicated a high proportion of participants were Indigenous and that there was a general lack of understanding amongst participants about standard drink sizes, a modified version of the original AUDIT-C wording was used, which has proven to be acceptable to Indigenous Australians [33,34]. The third item of the AUDIT-C, which refers to heavy drinking, was also modified to reflect the Australian Alcohol Guidelines in place at the time of this study [35] (see Table 1 for relevant modifications). Responses were scored as 0–4 and summed to a total ranging from 0 to 12. Risky drinking was measured using the validated Indigenous-specific AUDIT-C cut-off scores, and defined as a score of ≥5 [33]. For smoking, respondents were asked if they were current, occasional, ex-, or non-smokers (SP item). Current smokers were also asked the two item Heaviness of Smoking Index (HSI): how many cigarettes per day do you smoke; and how soon after waking do you smoke your first cigarette [36]. Using the standard classifications, a HSI score of 5 or more indicated high dependence. To measure illicit drug use, an abbreviated version of the illicit drug use questions in the Alcohol, Smoking and Substance Involvement Screening Test (ASSIST) questionnaire [37] was developed to achieve a balance between standardisation and response burden. The eight ASSIST illicit drug questions (which ask about specific drug use) were summarised into five: “Have you ever used cannabis”, and if yes, “How often did you use cannabis in the past three months?”, “Have you ever used an illicit substance (that was not cannabis)?”, and if yes, “How often did you use an illicit substance in the past three months that was not cannabis?”, and “which illicit substance did you use that was not cannabis?”

*Crime (N):* Respondents were asked if they had ever committed a crime, had ever been a victim of crime, had a high risk of exposure to crime in the home (defined as having lived with someone who had ever been to prison, or having lived with someone who had been released from prison in the past 6 months), and had they ever been involved with the juvenile justice system (defined as ever having to appear in court as the person of interest, or ever having been detained in a juvenile facility).

### 2.4. Procedure to Optimise the Feasibility of Applying Best-Evidence Measures

The new best-evidence assessment tool was designed with several practical features to facilitate flexible implementation in a dynamic program environment. First, it was developed to be delivered by staff in electronic format via tablet or laptop. These electronic devices pique participants’ interest in the assessment tool and allow staff to implement it across all three modes of service delivery. Second, it was designed so that it could be delivered in four discrete sections rather than requiring participants to complete the full assessment in one sitting. Third, a bespoke database was developed, into which assessment responses are automatically downloaded and from which pre-formatted reports can be generated. This allows staff to easily track participants’ progress over time and modify program activities to their changing needs. This process also allows researchers efficient access to de-identified data from consenting participants. Fourth, to ensure the ongoing utility of the assessment tool, electronic automatic reminders were built into the database to remind senior staff when follow-up assessments were due. This replicates computerised clinical decision support systems used in health care services that have improved the performance of health practitioners [38]. The program manager attended a one-day training session, facilitated by the researchers, on the new data collection procedures and management of the database. The manager then communicated this information to staff through their usual organisational processes.

For each participant, the intake procedure is initiated at the discretion of service staff, but it must occur within one month of their trial week to ensure risk factors are defined before behaviour change commences. Prior to commencing the intake procedure, participants are assured by staff that their responses are confidential. For young people who leave before qualifying as a participant but return at a later date, the intake procedure is re-initiated.

### 2.5. Statistical Methods

All analyses were performed using SPSS version 22 (SPSS Inc., Chicago, IL, USA) [39]. The feasibility of integrating best-evidence measures into the routine data collection system of the program was determined by the proportion of participants who completed the intake assessment and provided research consent. The proportion of missing data for each survey item was also measured. Frequencies and percentages for participants’ demographic characteristics and the different types of risk factors they report are presented as appropriate, except for participant resilience which is presented as a mean and median score. Participant age was aggregated into three categories (14 years, 15 to 18 years, and 19 to 21 years) based on anecdotal reports from the service staff that their participants were typically aged 15–18 years. Median age and the interquartile range for age were also reported. To calculate the number of risks experienced by each participant, a summary variable was created for each of the four domains of risk, based on positive endorsement of one or more of the risk factors within that domain. A simple count of the summary risk variables was calculated to represent the number of co-occurring risks experienced by each participant.

### 2.6. Ethical Considerations, Ethics Approval and Consent to Participate

All staff members are trained to screen for suicidal behaviours amongst participants. If, during administration of the assessment tool these behaviours are evident and the staff member feels the issue is beyond their training, the procedure is for the staff member to immediately stop the assessment, monitor their behaviour, and refer them on to a suicide call back service. If the participant does not want to talk to a staff member about this issue, and indicates he or she will not use the Suicide Call Back Service, the staff member will ask the young person for their permission to refer them to the School Counsellor, their local Headspace office, the community health centre, or other health professional qualified to address suicide-related issues.

Ethics approval (HC13055) was granted for this study by the University of New South Wales, University of New England, James Cook University, the University of Queensland, and the NSW Aboriginal Health and Medical Research Council. Participants were informed about the research study at the beginning of the formal intake procedure. They were assured that the inclusion of their data in the research was optional, that only de-identified data would be provided to the researchers from consenting participants, and that choosing not to provide consent would not impact their relationship with program staff, or their ability to attend the program. Only participants who provided signed consent (for participants 18 years or older), or assent and parental consent (for participants 17 years or younger), were eligible to participate in the study.

## 3. Results

### 3.1. The Feasibility of Embedding Data Collection in the Routine Processes of A Service for High-Risk Young People

**Figure 1:**
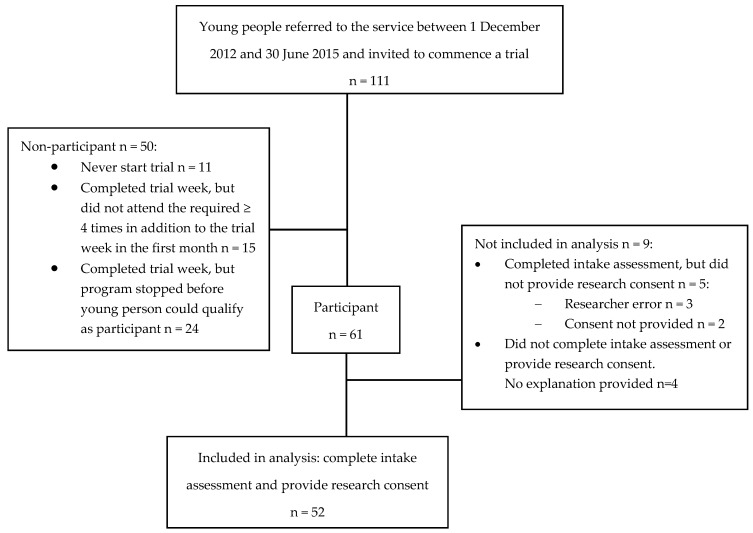
Flow-chart of referral to a service for high-risk young people.

As shown in Figure 1, 111 young people were referred to the service between 1 December 2012 and 30 June 2015 and invited to commence a trial. Of these, 50 (45%) did not go on to qualify as a participant: 11 (10%) did not accept the invitation to trial the program; 15 (14%) completed their trial week but did not attend the required four or more days in the first month; and 24 (22%) completed their trial week but could not attend the required four or more days in the first month because the program was stopped in their community. Of the participants (n = 61), nine were excluded from the analysis: three because, although completing the intake assessment, the correct research consent was not obtained due to researcher error; two because, although completing the intake assessment, they did not provide their research consent; and four because they did not complete the intake assessment or provide their research consent. The final study sample size was 52 (85%) of the 61 participants.

Of the 19 items relating to risk factors in the intake assessment, five items (26%) were completed by all 52 participants, and 16 items (84%) were completed by at least 80% (42 or more) of participants. Three measures of risk were completed by less than 80% of participants: (i) “How often have you used an illicit substance in the past three months?”; (ii) “Have you committed a crime?”; and, (iii) “Have you been a victim of crime?”.

### 3.2. Demographic Characteristics and Risk Factors of Program Participants

*Demographic characteristics*: As reported in Table 2, 89% of program participants were male, with the majority (91%) aged 15 to 18 years. The median age was 17 years. Indigenous young people were over-represented (49%) given the Indigenous population in the New England region of NSW is 9% [40]. A similar proportion of participants received the program in each of the three formats, ranging from 25%–42%.

*Education and employment*: Eighty-one per cent of participants had been suspended from school three or more times in the past six months, 23% reported that they did not usually attend school, 76% were unemployed, and 19% reported that they were receiving government financial benefits.

*Mental health and wellbeing*: Fifty-five per cent of respondents responded positively to at least one of the five suicide questions, placing them at-risk of suicide ideation, and the same proportion reported experiencing moderate mental distress in the past four weeks, whilst 10% reported serious mental distress. The mean and median resilience score was 24 out of a possible 40 (a recent study reported an average resilience score of 30 for U.S. youth populations) [27]. Twenty-seven per cent of participants reported eating fast food three or more times in the past week, 24% reported doing no exercise in the past week, and 79% had not visited a health professional for more than one year.

*Substance use:* Approximately two-thirds of respondents (65%) reported risky drinking according to the Indigenous specific AUDIT-C cut-off scores. Seventy-five per cent of respondents reported being a current cigarette smoker and 18% were highly tobacco dependent. Three quarters of respondents had ever tried an illicit substance (67% had only ever tried cannabis), and 38% reported at least weekly use of an illicit substance (including cannabis) in the past three months. Of these, approximately four times as many respondents reported using cannabis (34%) compared to any other illicit substance (9%). Of the respondents who did report using an illicit substance other than cannabis in the past three months, use of speed and prescription medications was reported.

*Crime*: Sixty-two per cent of respondents reported ever having committed a crime, 38% reported ever being the victim of a crime, 43% reported exposure to crime in the home, and 40% reported having been involved with the juvenile justice system.

*Co-occurring risk*. Ninety-eight per cent (n = 51) of participants experienced two or more co-occurring domains of risk, the median number of domains of risk experienced by participants was four, and 58% of participants experienced co-occurring risk in all four domains.

## 4. Discussion

### 4.1. The Feasibility of Embedding Data Collection in the Routine Processes of a Service for High-Risk Young People

This study demonstrates that standardised, methodologically rigorous data on participant risk factors can feasibly be collected by embedding a best-evidence assessment tool into the routine data collection processes of a service for high-risk young people: of the 61 program participants in a 19-month period, 52 (85%) completed the intake assessment, and the majority (84%) of survey items were completed by 80% of participants. The majority of program participants were male and aged 15 to 18 years, and Indigenous participants (49%) were clearly over-represented relative to their population (9%) [40]. All but one participant experienced at least two co-occurring domains of risk, and the majority of participants (58%) experienced co-occurring risk across all four domains. The most prevalent risk factors were frequent school absence (whether voluntarily or because they had been suspended), unemployment, a propensity towards suicidality, high levels of mental distress, weekly illicit substance use, risky drinking, smoking, low levels of physical activity, low utilisation of health services, involvement in crime, exposure to household members with a history of incarceration, and involvement with the juvenile justice system.

### 4.2. The Utility of Embedding Data Collection in the Routine Processes of a Service for High-Risk Young People

The results from this study have a number of implications surrounding the utility of the data, particularly in relation to service delivery.

First, given the benefits of tailoring interventions to the specific risk factors of participants [41,42], by demonstrating that it is feasible for services to collect rigorous data on risks experienced by their participants services are provided with an opportunity to tailor their current activities to improve the precision with which they target the most prevalent risk factors experienced by participants. For example, some of the more serious risks identified among participants of this particular program, such as mental distress and suicide ideation, suggest that providing access to evidence-based therapies such as Cognitive Behaviour Therapy and Motivational Interviewing, as well as a suicide-specific risk assessment and response tools, such as the Suicide Assessment Kit (SAK) [43], should be a priority across all program components. Similarly, as substance use, crime, and exposure to incarceration among household members were found to be problematic, the core program component that focuses on diversionary activities could be expanded to offer emergency accommodation which is safe and secure, and has ready access to highly qualified staff. This would further reduce participants’ exposure to high-risk people and situations and may reduce their levels of mental distress.

In addition to poor mental health and wellbeing, participants were found to experience risks associated with poor physical health. A tailored health and wellbeing component, which includes activities emphasising the importance of accessing and preparing nutritious food, engaging in regular exercise and having access to health professionals could be an important addition to the program. These activities could include cooking demonstrations, shopping tours that provide guidance on how to obtain relatively inexpensive, nutritious food, group exercise classes or dedicated personal training sessions, and on-site health checks delivered in partnership with local General Practitioners and/or Aboriginal Medical Services. Given the finding that 75% of participants were current cigarette smokers, two-thirds were risky drinkers and 38% used illicit substances on a weekly basis, on-site health checks would also provide opportunities for substance use cessation intervention. The over-representation of Indigenous young people in these data also suggest that all program components should include activities which emphasise the importance of, and facilitate meaningful access to, Indigenous culture, elders and traditional country.

Second, in addition to facilitating tailoring when participants commence a service, these service-specific data allow staff to monitor participant risk factors, through re-administration of the assessment tool at regular time intervals, ensuring a mechanism for the service to adapt to the changing needs of participants, whilst also providing an opportunity for staff to provide personalised feedback to participants to motivate them to maintain their change in risk behaviour [44].

Third, these regularly collected data provide an opportunity for services to measure the effectiveness of their programs whilst adjusting for baseline risk. This could be readily undertaken by research partners based in local, regional universities or in major metropolitan universities. Alternatively, the service could hire the services of a statistician, or employ an administrative person to conduct basic analyses in Microsoft Excel, which would impose minimum expense to the service.

A complementary benefit of having identified a best-evidence assessment tool that can be feasibly integrated into the routine processes of a service for high-risk young people, is that it is likely to improve the consistency with which similar programs measure outcomes, increasing opportunities to pool results to draw conclusions about the effectiveness of programs for high-risk young people.

### 4.3. Other Implications of This Study

The finding that nearly one quarter of participants are usually absent from school (23%) highlights the need for community-based services that can effectively engage with this small number of vulnerable young people, given they are unlikely to access programs offered through schools or other educational institutions. This finding also reinforces the importance of the skills and learning core component of this program in ensuring young people achieve at least a basic level of education.

These results also show that despite participants representing only 0.5% of young people in the region where this study was conducted, they contribute to a high proportion of crime in their communities: 62% report having committed a crime; 43% report being exposed to crime in the home; and 40% report having been involved with the juvenile justice system. This finding suggests there is scope for future studies to examine routinely collected, unit-level crime data to determine whether services for high-risk young people have an impact at the community level through reducing the incidence of youth crime and anti-social behaviour.

Finally, this study points to the utility of a strict referral procedure into the service (detailed in the Methods section), which ensures a replicable and largely objective process of referral into the service, and minimises inefficient allocation of resources to young people who are unlikely to benefit from participation in the program. For this program, the well-defined referral procedure meant only 61 of the 111 young people referred accessed a substantial part of the program, 85% of whom completed the assessment and engaged for at least four weeks. Future evaluation of this and similar programs could establish retention rates after three, six or twelve months to further gauge the success of the intake procedure in specifically engaging with those young people who are most likely to benefit from these types of community-based programs.

### 4.4. Limitations

Although it is possible that not all risk factors relevant to high-risk young people have been captured in this assessment tool, it does reflect the combined knowledge of the service providers and researchers. Nevertheless, it may need to be revised to ensure it is relevant to other services for high-risk young people delivered in different settings. As was done for the intervention itself, the tension between standardising and tailoring measures could be resolved by establishing a toolbox of core measures for particular risk factors, which all services working with high-risk young people could utilise for their intake assessments. This standardised intake assessment could then be augmented with tailored measures that are of particular interest to service staff.

As the assessment tool was developed as part of a collaborative effort with program staff, trade-offs were made to reduce reporting burden on participants and to ensure that it was feasible to deliver for staff. This meant that, evidence-based assessment items were selected where available, but that it was necessary for items with no evidence-base, or with no evidence-base for young people, were developed where required. Future research could usefully establish the reliability and validity of these new assessment items for this particular sub-population of young people. Additionally, in some cases, modifications were made to existing evidence-based measures to reduce respondent burden. The ASSIST (Alcohol, Smoking and Substance Involvement Screening Test) is one such example. Although this appears to have been appropriate for this service, given the small proportion of participants who reported using illicit substances other than cannabis, it may not be appropriate for other services as it could result in the under-reporting of particular types of illicit substance use. Given the current concern surrounding methamphetamine use in Australia, for example, and evidence to suggest that the number of regular and dependent users in recent years has risen, particularly among young people aged 15 to 34 years [45], the full version of the ASSIST might be more suitable for future iterations of this assessment tool. Similarly, despite using the modified wording of the AUDIT-C, which has proven acceptable to Indigenous people, further study is required to establish the reliability and validity of these questions for high-risk young people. There is also a need to determine whether using open-ended responses for the AUDIT-C questions provides a measure of alcohol risk status that has comparable reliability and validity to the standard categorical responses in AUDIT-C that are based on the concept of standard drinks, particularly as Indigenous Australians are unlikely to conceptualise their drinking in those terms [46]. In practical terms, this is also an important consideration because open-ended questions would eliminate the need to modify the assessment tool when national guidelines [35] are updated, but response options for standardised measures, such as the AUDIT-C, remain unchanged [47].

The measures of risk that were completed by less than 80% of participants, specifically those relating to illicit substance use in the past three months, participation in crime and being a victim of crime, could have led to an under-, or over-representation of risk in the domains of substance use and/or crime. It also signals that participants might have been uncomfortable responding to these items. In future, instead of using a self-report intake assessment to collect baseline data on these risk factors, perhaps objective measures would be more appropriate (e.g., gaining permission to access participants’ routinely collected, deidentified, police incident data). Obtaining this type of data would require safeguarding the identity of participants to ensure analysis was not conducted by someone with intimate knowledge of participants’ histories (because this could allow re-identification even without names), such as the research partner or the statistician. This would limit the ability to use these data for tailoring program activities to individual participant needs, but it would still be useful for overall program evaluation and identification of program priorities.

Finally, given staff in this service anecdotally reported that the intake assessment took some time to complete and was usually not their priority when faced with participants exhibiting difficult behaviour, an abbreviated version of the assessment tool could be developed using standard psychometric methods to further improve the feasibility of integrating best-evidence measures into the routine data collection processes of the service. Leveraging the partnership developed during the co-creation process, a further solution could be to have researchers deliver basic training to program staff in research methods to improve their understanding of the importance and benefits of rigorous and systematic data collection.

## 5. Conclusions

As highlighted by an earlier systematic review of the international literature, this is the first study to demonstrate that best-evidence measures can feasibly be embedded into the routine data collection processes of a service for high-risk young people [15]. Replication of this process in other services would improve the quality of services available for high-risk young people, facilitate a greater number of high quality evaluations of these services, and contribute much needed evidence on how to improve outcomes for high-risk young people.

## Figures and Tables

**Table 1:** AUDIT-C—adapted wording for Indigenous Australians.

Adapted AUDIT-C Item	Original AUDIT-C Item	Response	Score
1. How often do you have a drink of alcohol?	How often do you have a drink containing alcohol?	Never	0
		Monthly or less	1
		2–4 times a month	2
		2–3 times a week	3
		4 or more times a week	4
2. When you have a drink of alcohol, how many drinks do you usually have?	How many standard drinks containing alcohol do you have on a typical day when drinking?	1 or 2	0
		3 or 4	1
		5 or 6	2
		7 to 9	3
		10 or more	4
3. How often do you have five or more drinks all in one go?	How often do you have six or more drinks on one occasion?	Never	0
		Less than monthly	1
		Monthly	2
		Weekly	3
		Daily or almost daily	4

**Table 2:** Demographic characteristics and risk factors of participants.

Characteristics	Participants (n = 52)		
	N	Total	%
Demographics			
Sex: Male	46	52	89
Age (years):			
14	2	45	4.5
15–18	41	45	91
19–21	2	45	4.5
Median (IQR)	17 (2)		
Identify as Indigenous	23	47	49
Communities of residence clustered by the service delivery site:			
Community 1 (on-site program based in shed)	17	52	33
Communities 2 and 3 (outreach program)	13	52	25
Communities 4 and 5 (on-site program based on farm)	22	52	42
Risk domain 1: Education and employment			
Suspended ≥3 times in past 6 months	39	48	81
Do not usually attend school	10	43	23
Unemployed	39	51	76
Receive government financial benefit	10	52	19
Risk domain 2: Mental health and wellbeing			
Experienced suicide ideation in past 4 weeks	26	47	55
Experienced moderate mental distress in past 4 weeks	28	51	55
Experienced serious mental distress in past 4 weeks	5	51	10
Resilience: Mean (Median)	24 (24)	48	
Ate fast food ≥3 times in past week	14	52	27
Do not exercise in past week	12	51	24
Last visit to health professional ≥1 year	38	48	79
Risk domain 3: Substance use			
Risky drinker	33	51	65
Current smoker	39	52	75
HSI: High tobacco dependence	9	51	18
Have tried illicit substances (including cannabis)	38	50	76
At least weekly illicit substance use in past 3 months	15	40	38
Risk domain 4: Crime			
Have committed a crime	23	37	62
Have been a victim of crime	13	34	38
High risk of exposure to crime in the home	20	46	43
Have been involved with the juvenile justice system	17	43	40

## Abbreviations

The following abbreviations are used in this manuscript:

ASSIST	Alcohol, Smoking and Substance Involvement Screening Test
AUDIT	Alcohol Use Disorders Identification Test
BE	Best-evidence
CD-RISC-10	Connor Davidson Resilience Scale (10-item)
HSI	Heaviness of Smoking Index
K6	Kessler Psychological Distress Scale (six-item)
N	New item
NSW	New South Wales 0
SP	Similar population group
SAK	Suicide Assessment Kit
